# The Virtual Man Project’s CD-ROM “Voice Assessment: Speech-Language Pathology and Audiology & Medicine”, Vol.1

**DOI:** 10.1590/S1678-77572009000700008

**Published:** 2009

**Authors:** Millena Maria Ramalho Matta VIEIRA, Giédre BERRETIN-FELIX, Alcione Ghedini BRASOLOTTO

**Affiliations:** 1 BS, Speech-Language pathologist; 2 BS, MSc, PhD, Assistant Professor of the Department of Speech-Language Pathology and Audiology, Bauru School of Dentistry, University of São Paulo, Bauru, SP, Brazil

**Keywords:** CD-ROM, Distance education, Assessment, Voice

## Abstract

The CD-ROM “Voice Assessment: Speech-Language Pathology and Audiology & Medicine” was developed as a teaching tool for people interested in the production of the spoken or sung human voice. Its content comprises several subjects concerning the anatomy and physiology of spoken and sung voice. A careful assessment becomes necessary in order to ensure the effectiveness of teaching and learning educational materials, whether related to education or health, within the proposal of education mediated by technology. Objective: This study aimed to evaluate the efficacy of the Virtual Man Project’s CD-ROM “Voice Assessment: Speech-Language Pathology and Audiology & Medicine”, as a self-learning material, in two different populations: Speech-Language Pathology and Audiology students and Lyrical Singing students. The participants were instructed to study the CD-ROM during 1 month and answer two questionnaires: one before and another one after studying the CD-ROM. The quantitative results were compared statistically by the Student’s t-test at a significance level of 5%. Results: Seventeen out of the 28 students who completed the study, were Speech-Language Pathology and Audiology students, while 11 were Lyrical Singing students (dropout rate of 44%). Comparison of the answers to the questionnaires before and after studying the CD-ROM showed a statistically significant increase of the scores for the questionnaire applied after studying the CD-ROM for both Speech-Language Pathology and Audiology and Lyrical Singing students, with p<0.001 and p<0.004, respectively. There was also a statistically significant difference in all topics of this questionnaire for both groups of students. Conclusion: The results concerning the evaluation of the Speech-Language Pathology and Audiology and Lyrical Singing students’ knowledge before and after learning from the CD-ROM allowed concluding that the participants made significant improvement in their knowledge of the proposed contents after studying the CD-ROM. Based on this, it is assumed that this didactic material is an effective instrument for self-learning of this population.

## INTRODUCTION

The use of information technology in the areas of education and health with teaching and assistance purposes has continuously increased. Technology-assisted education is a teaching modality that employs didactic resources, presented as different information bases. It can involve several means of communication facilitating the self-learning process^8^ and the students’ independence because they will be able to study according to their own capacity, anywhere and at any time^12^. The use of these pedagogical learning strategies, also denominated as collaborative strategies, is very effective to increase the motivation level of the participants and for the accomplishment of the proposed activities^6^.

Under the coordination of the Professor Chao Lung Wen, the Virtual Man Project is part of the category of learning goals of the Medical School of the University of São Paulo (FM/USP), described as a powerful iconographic resource that helps learning, considering that it facilitates understanding in relation to a specific subject^3^. As a result of a partnership among the Medical School of the University of São Paulo (FM/USP), Bauru School of Dentistry of the University of São Paulo (FOB/USP) and Federal University of São Paulo (UNIFESP), a CD-ROM denominated “Voice Assessment: Speech-Language Pathology and Audiology & Medicine”, vol. 1, was developed under the coordination of Professor György Mikϵlós Böhm (FM/USP). This volume comprises the following subjects: upper commands and innervations of the phonation tract; functional anatomy of the larynx; physiological functions of the larynx; vocal tract (forming and vibrating); sound articulation (vowels and consonants) and singing. It is a kind of technology that allows observing the speech tract in a three-dimensional and dynamic way, enabling the comprehension of the speech complexity in a simpler way. In addition to the iconographic communication of the Virtual Man Project, the CD-ROM contains sounds, films, several illustrations and texts^16^. This material has been developed to be applied as a didactic instrument for both undergraduate and graduate students, and anyone interested in the production of the human voice, spoken or sung.

The elaboration of a didactic material of any nature always demands previous analysis in order to promote actual learning facilities to the students^15^. The evaluation of an educational software, for example, is a step of fundamental importance for reaching the proposed goals and comprise the teaching-learning problem that has motivated its creation^17^.

The aim of the present study was to analyze the efficacy of the Virtual Man Project’s “Voice Assessment: Speech-Language Pathology and Audiology & Medicine”, vol. 1, as a self-learning material for two distinct populations.

## MATERIAL AND METHODS

The present study was reviewed and approved by the Department of Speech-Language Pathology and Audiology and by the FOB/USP’s Research Ethics Committee (Process #120/27). The participants received verbal and written explanations about the study purposes and signed an informed consent form.

Thirty students attending the 1^st^ semester of the Speech-Language Pathology and Audiology course at FOB/USP and 30 students attending the 1st-6th year of the Lyrical Singing course at the Drama and Music Conservatory “Dr. Carlos de Campos”, in the city of Tatuí, SP, were invited to participate. At first, 50 students accepted taking part in the study, being 23 Speech-Language Pathology and Audiology students and 27 Lyrical Singing students. The following exclusion criteria were adopted: previous contact with the content of the CD-ROM under study; and not having access to a computer with CD reader and without specific softwares for images and sounds.

Initially, a questionnaire denominated “before CD-ROM questionnaire” containing one open and multiple-choice questions was handed to the students. All questionnaires had 4 options of answers (a, b, c and d), the last one being “I do not know the answer”. The questionnaires were different according to each group. For the Speech-Language Pathology and Audiology students, 44 questions were elaborated about the following topics of the CD: upper commands and innervation of the phonation tract (17 questions), functional anatomy of the larynx (7 questions), physiology of the larynx (14 questions) and human voice sound articulation (6 questions). For the Lyrical Singing students, 36 questions were elaborated about the following topics of the CD: upper commands and innervation of the phonation tract (6 questions), functional anatomy of the larynx (7 questions), physiology of the larynx (14 questions) and sung voice (8 questions).

All students received a Virtual Man Project’s “Voice Assessment: Speech-Language Pathology and Audiology & Medicine”, vol. 1, and an auxiliary study guide, which was elaborated with some questions related to the topics of the CD for both courses. The Speech-Language Pathology and Audiology students were oriented to study the following subjects: upper commands and innervations; propositional vocalization, emotional vocalization, important skull nerves for phonation, larynx (functional anatomy: cartilages, ligaments and articulations; extrinsic muscles; intrinsic muscles, vocal fold; glottis configuration), physiology of the larynx (functions of the larynx, phonation, types of voice, vocal tract (forming and vibrating), human voice (sound articulation: vowels and consonants). The Lyrical Singing students were oriented to study the following subjects: larynx (vocal fold, glottis configuration) physiology of the larynx (functions of the larynx, phonation, types of voice, vocal tract (forming and vibrating) singing (phonation mechanisms and some aspects of sung voice).

After answering the “before CD-ROM questionnaire” and receiving the didactic material (CD-ROM and the auxiliary study guide), a period of 1 month was granted to the participants to study. After that period, the participants were contacted in person or by e-mail or telephone to schedule a date for answering a second questionnaire, denominated “after CD-ROM questionnaire”. This second questionnaire was elaborated with the exact same questions of the “before CD-ROM questionnaire” and some other open questions, which aimed at knowing the opinions and suggestions of the students about the CD-ROM as well as any problems they found during the study.

As a strategy for the students to study the material within the established period, they were informed that the FOB/USP’s Department of Speech-Language Pathology and Audiology would provide them an attendance certificate at the end of the research and another certificate of good performance for the ones who reached the score of 75% or higher on the “after CD-ROM questionnaire”.

The t-test for dependent samples was applied for analysis of the quantitative results (comparison of the answers of the questionnaires applied before and after the CD-ROM), as it was considered significant p<0.05. An analysis of the qualitative results was performed by means of questions referring to the participants’ suggestions and the problems they faced when studying the CD-ROM.

## RESULTS AND DISCUSSION

From the 50 students that initially accepted taking part in the study, only 28 (56%) remained until the end of the investigation. From these 28 participants, 17 (60.71%) were Speech-Language Pathology and Audiology students and 11 (39.28%) were Lyrical Singing students. There were only four male students, one from the Speech-Language Pathology and Audiology course and three from the Lyrical Singing course. The mean ages were 18.94 and 27.72 years for the Speech-Language Pathology and Audiology and Lyrical Singing students. The dropout rate was 44%, being 16 students who from the Lyrical Singing course and 6 students from the Speech-Language Pathology and Audiology course. The students who retired from the study alleged that they did not have enough time to study the CD-ROM. Although the study of the CD-ROM has not been proposed as a formal course, the results are in agreement with the literature, which points a dropout rate around 50%^5^^,^^13^,^14^ for technology-assisted educational courses, lack of time being the most frequently reported reason for giving up.

Considering that the maximum score to be reached by the Speech-Language Pathology and Audiology students on the “before CD-ROM questionnaire” was 44 points, the mean of correct answers of all students was 5.94 (13.35%), with of minimum of zero and maximum of 12 points (27.27%). On the “after CD-ROM questionnaire”, the students had 20.59 (46.79%) mean of correct answers, with minimum of 11 (25%) and maximum of 39 points (88.63%). Comparison of the results of both questionnaires showed a statistically significant increase (p<0.001) on the scores of the “after CD-ROM questionnaire”. Statistically significant differences were also present in all topics of this questionnaire (Table 1).

**Table 1:** Results before and after CD-ROM study, by topics – Speech-Language Pathology and Audiology students

Topics	Mean	SD	Min.	Max.	T	p
Upper Commands - Before	1.71	1.4	0	5	- 7.217	<0.001*
Upper commands - After	7.71	3.46	3	17		
Functional Anatomy - Before	1.06	1.03	0	3	- 5.66452	<0.001*
Functional Anatomy - After	3.59	1.58	1	7		
Larynx Physiology - Before	2.12	1.69	0	5	- 5.87169	<0.001*
Larynx Physiology - After	6.24	2.82	2	11		
Sound Articulation - Before	0.94	1.14	0	3	- 6.96262	<0.001*
Sound Articulation - After	3.24	1.25	1	6		
Total Before	5.94	3.49	0	12	- 7.71699	<0.001*
Total After	20.59	7.3	11	39		

* Statistically significant difference (p<0.05)

The results of the “before CD-ROM questionnaire” for the Lyrical Singing students, which had as maximum value 36 points, the mean of correct answers was 6,45 (17,91%), with minimum of 1 (2.77%) and maximum of 16 points (44.44%). On the “after CD-ROM questionnaire”, the mean of correct answers was 15.73 (43.69%), with minimum of 1 (2.77%) and maximum of 29 points (80.55%). Comparison of the results of both questionnaires showed a statistically significant increase (p<0.004) on the scores of the “after CD-ROM questionnaire”. Statistically significant differences were also present in all topics of this questionnaire (Table 2).

**Table 2:** Results before and after CD-ROM study, by topics – Lyrical Singing students

Topics	Mean	SD	Min.	Max.	T	p
Upper Commands - Before	0.82	0.75	0.00	2.00	-4.3788	<0.001*
Upper commands - After	2.73	1.10	1.00	5.00		
Functional Anatomy - Before	0.64	0.81	0.00	2.00	-4.98098	<0.001*
Functional Anatomy - After	3.18	1.54	0.00	5.00		
Larynx Physiology - Before	2.64	1.69	1.00	6.00	-2.29661	<0.045*
Larynx Physiology - After	5.45	3.59	0.00	12.00		
Sung Voice Before	2.18	2.40	0.00	6.00	-2.49723	<0.032*
Sung Voice After	4.55	2.38	0.00	9.00		
Total Before	6.45	4.48	1.00	16.00	-3.75671	<0.004*
Total After	15.73	7.42	1.00	29.00		

* Statistically significant difference (p<0.05)

It is possible to notice that the maximum score for the “before CD-ROM questionnaire” reached by the Speech-Language Pathology and Audiology students was similar to the minimum reached for the “after CD-ROM questionnaire”. Similarly, better results were observed in the “after CD-ROM questionnaire” by the Lyrical Singing students when compared to the “before CD-ROM questionnaire”, demonstrating the effectiveness of the study also for this group. Through a written evaluation of a study proposal employing the CD-ROM, statistically significant differences were also observed before and after the test, demonstrating that the elaborated material helped the student learning2. A study that examined the impact of an online course on healthcare professionals’ competency in infection prevention using questionnaires applied before and after the course for knowledge evaluation, concluded that the participants made statistically significant increases in their perceptions of competency in infection control following the course. Additionally, most of the participants were very pleased and reported that the acquired knowledge was useful and relevant for their professional practice^1^.

For the Speech-Language Pathology and Audiology students, the topic in which there were more correct answers in the “before and after CD-ROM questionnaires” was articulation of the human voice sounds (before = 16.66% and after = 52.94%). The topics that presented the largest number of incorrect answers in the “before CD-ROM questionnaire” were functional anatomy of the larynx and physiology of the larynx (84.87%). In the “after CD-ROM questionnaire”, the topic that presented more incorrect answers was physiology of the larynx (55.46%). The option “I do not know the answer” was employed more frequently on the topic upper commands and innervation of the phonation tract (73.35%) in the “before CD-ROM questionnaire” and on the topic physiology of the larynx (18.48%) in the “after CD-ROM questionnaire”.

For the Lyrical Singing students, the topic that presented more correct answers in the “before and after CD-ROM questionnaires” was sung voice (before = 24.24% and after = 49.49%). The topic that presented the largest number of incorrect answers in the “before CD-ROM questionnaire” was functional anatomy of the larynx (89.61%). In the “after CD-ROM questionnaire”, the topic that presented more incorrect answers was physiology of the larynx (61.03%). The option “I do not know the answer” was employed more frequently on the topic functional anatomy of the larynx in both the before (80.51%) and after (24.02%) CD-ROM questionnaires.

The mean of correct answers for the Speech-Language Pathology and Audiology students in the “after CD-ROM questionnaire” was 20.59 and for the Lyrical Singing students was 15.75. It was observed a relevant improvement of 14.65 points for the Speech-Language Pathology and Audiology students and 9.30 points for the Lyrical Singing students. It appears that the items that presented the largest number of correct answers for both groups were those that were directly related to their area of study.

It was observed that one Lyrical Singing student presented more correct answers in the “before CD-ROM questionnaire” (2 correct) than “after CD-ROM questionnaire” (1 correct). In addition, another student presented the same results for before and after the CD-ROM (16 correct answers). This was not observed for the Speech-Language Pathology and Audiology students. Educational courses mediated by technology require mature, self-confident and self-motivated students that are normally better applied to professionals who are already working and intend to be successful in their careers or are constantly being required for updating knowledge^7^. It is known that motivation is a determining factor for the success of the learning process. In addition, it is important that the students organize their time and space for studying, as well as developing autonomous learning strategies, so that they may become the active subject of their cultural background, developing the learning process in any environment^4^.

Referring to the frequency of the study of the CD-ROM, most students of both groups reported that they studied from 3 to 4 times the material. Although a quantitative analysis was not performed for such purpose, the score reached by the students in relation to the frequency of the study indicates that it did not interfere on the performance of the students, because the ones who had the higher frequency of study were not the ones who presented the highest scores.

In the beginning of the “after CD-ROM questionnaire”, there were questions referring to the CD-ROM use itself as well as space for the participants presenting suggestions and/or problems faced during the study. For the questions regarding to static and dynamic images, most of the answers were Very good and Excellent. About the texts of the CD-ROM, most answers were Very good. There were no answers Poor or Regular for those items. About the theoretical content, most of the evaluations were Good and Very good, and there was no Poor evaluation.

When the students were asked if they faced any difficulty in exploring the CD-ROM, only 2 (11.76%) Speech-Language Pathology and Audiology students and 4 (36.36%) Lyrical Singing students answered affirmatively. The problems were related to the computer, for example: incompatible programs for the CD-ROM, “slow computer”, bad functioning of the videos, and computer technical failure.

Regarding the question on the problems faced about the theoretical content of the CD-ROM, 1 (5.88%) Speech-Language Pathology and Audiology student did not answer the question; 5 (29.41%) Speech-Language Pathology and Audiology students and 7 (63.63%) Lyrical Singing students reported that they did not face any problems. The problems reported by the participants referred to the type of vocabulary contained in the CD-ROM, which was considered very technical and specific; the immaturity of the students to understand complex subjects for being students of the first year of college, and some topics that were too long.

From a total of 17 Speech-Language Pathology and Audiology students, only 7 (41.17%) gave some suggestions for the CD-ROM and from the 11 Lyrical Singing students, only 6 (54.54%) gave some suggestions. The suggestions given by the Speech-Language Pathology and Audiology students were: “It would be better if the videos were a little slower”; “Make them accessible on Windows Vista”; “Although the texts were narrated before the videos, it would be interesting to have a narration during the videos”; “More pictures are necessary, so that the texts can be more explored”; “Pronunciation of just the sound of the phoneme, without the sound of the consonants”; “Make the CD run in other programs”. The suggestions given by the Lyrical Singing students: “Employ a simple language and explain the technical terms”; “Less texts and more explanations presenting the tables, more graphics and clips, all simultaneously”; “Allow another type of access as DVD”; “Elaborate an easier language”; “Add more explanations about respiration”; “Add more pictures”.

Only 2 students, one from each group, obtained a score higher than 75% in the “after CD-ROM questionnaire”. The Speech-Language Pathology and Audiology student obtained 88.63% score and the Lyrical Singing student 80.55%.

Overall, the aspects related to static and dynamic images, texts and theoretical content were positively evaluated by the students. Similarly, the subjective evaluation of the participants of a multimedia program about pleural drainage^10^ presented some results like excellent, very good and good, concerning the aspects related to informatics and content. Another study about the development and evaluation of a multimedia system about irrigation^9^ required 56 people to evaluate a CD-ROM in three different ways: as a whole, on the didactic aspect and only one chapter of the CD-ROM. The educational role of the software was evaluated in relation to the facility to access to the screen; the sequence of the units of the CD-ROM; the amount of information; the quality of the videos, audio, digital pictures, graphic animation and written information; if the examples presented were important for the student to learn; if the material corresponded to the expectations of the user in both theoretical and practical terms. The evaluation indicated that the developed multimedia system may be applied as a didactic resource for teaching and learning purposes^9^.

There is no doubt that it is necessary to evaluate all the possible aspects of the materials that are employed for teaching and learning, whether they are concerned to health or education. Thus, studies must regard not only the elaboration and creation, but also the evaluation of the material. The follow up during all steps of the study of the CD-ROM by the learner may reduce the motivational problems found and allows for expanding the knowledge about its use.

An aspect that may contribute for understanding the ability of learning from the use of the CD-ROM refers to its usability, and this should be addressed in further studies. Usability is a set of factors that ensure that the products are easy to manage, efficient and pleasant, according to the perspective of the user^11^. Employing some strategies to evaluate usability enables the comprehension for some ways of learning for each user and also the difficulties faced during the process. Although it has not been possible to employ usability strategies on the present study, an auxiliary guide with questions referring to the CD-ROM topics was supplied to help students organizing themselves. In addition, the students were free to contact the researcher for clarification about the use of the CD-ROM.

Another very important aspect of this investigation was having two distinct groups evaluating the CD-ROM, employing traditional methodologies and modern methodologies that use the technological resources in order to compare the ways students study and learn, as well as the teaching methods.

The teaching tool evaluated in this investigation contributes to the knowledge of some subjects referring to the assessment of voice, its upper commands and innervation of the larynx, anatomy and physiology, articulation of the voice sound and sung voice. The findings of the present investigation contributed to validate the efficacy of this didactic material for the population under study and this CD-ROM was proven useful for both in loco and at distance teaching modalities. Diverse populations can benefit from this material, including doctors, healthcare and education professionals, ordinary people, students and even patients.

## CONCLUSION

The results concerning the evaluation of the Speech-Language Pathology and Audiology and Lyrical Singing students’ knowledge before and after learning from the CD-ROM allowed concluding that the participants made significant increase in their knowledge of the proposed contents after studying the CD-ROM. Based on this, it is assumed that this didactic material was an effective instrument for self-learning of this population.
